# Fretting Wear Behavior of Three Kinds of Rubbers under Sphere-On-Flat Contact

**DOI:** 10.3390/ma14092153

**Published:** 2021-04-23

**Authors:** Tengfei Zhang, Jie Su, Yuanjie Shu, Fei Shen, Liaoliang Ke

**Affiliations:** 1 School of Mechanical Engineering, Tianjin University, Tianjin 300350, China; tfzhang@tju.edu.cn (T.Z.); jiesu@tju.edu.cn (J.S.); shenfei@tju.edu.cn (F.S.); 2 Institute of Engineering Mechanics, Beijing Jiaotong University, Beijing 100044, China; 17115274@bjtu.edu.cn

**Keywords:** rubber, fretting wear, wear mechanism, wear volume

## Abstract

Rubbers are widely used in various fields as the important sealing materials, such as window seal, door seal, valve, pump seal, etc. The fretting wear behavior of rubbers has an important effect on their sealing performance. This paper presents an experimental study on the fretting wear behavior of rubbers against the steel ball under air conditions (room temperature at 20 ± 2 °C and humidity at 40%). Three kinds of rubbers, including EPDM (ethylene propylene diene monomer), FPM (fluororubber), and NBR (nitrile–butadiene rubber), are considered in experiments. The sphere-on-flat contact pattern is used as the contact model. The influences of the displacement amplitude, normal force, frequency, and rubber hardness on the fretting wear behavior are discussed in detail. White light profiler and scanning electron microscope (SEM) are used to analyze the wear mechanism of the rubber surface. The fretting wear performances of three rubbers are compared by considering the effect of the displacement amplitude, normal force, frequency, and rubber hardness. The results show that NBR has the most stable friction coefficient and the best wear resistance among the three rubbers.

## 1. Introduction

Rubber is a kind of soft material widely used in daily life, aerospace, construction machinery, transportation, and other fields [1,2,3], such as soft crawling robots, mechanical seal gaskets, car windows, etc. It possesses high structural flexibility, good environmental adaptability, strong affinity, and low price, and thus has very broad applications. During the application of rubbers, they will inevitably come into contact, friction, and wear with surrounding objects and even themselves. Especially, when they work in a vibrational environment, the fretting wear will occur at the contact surfaces of rubbers, which reduces the sealing performance and reliability of rubber materials. Therefore, the evaluation of the sealing performance of rubbers in engineering fields is of importance.

Fretting refers to the relative movement of two contact surfaces with very small amplitude [4], which is generally on the order of microns. Fretting wear refers to the wear on the contact surface caused by the small relative displacement of the contact pair subjected to local contact load or fixed prestress due to the external vibration. The fretting can cause friction and wear on the contact surface, resulting in looseness, power loss, noise increase or pollution formation, etc. Fretting can also accelerate the initiation and expansion of fatigue cracks and greatly reduce the fatigue life of components. At present, there is little research on micron-level fretting wear (displacement amplitude <1 mm) of rubber materials. The related studies mainly focus on the fretting wear with a relatively large displacement amplitude (displacement amplitude ≥1 mm). Baek and Khonsari [5,6,7] systematically studied the friction and wear characteristics of rubber coatings with the effect of normal force, velocity, displacement amplitude, temperature, and surface roughness. Karger-Kocsis et al. [8,9] studied the friction and wear performances of ethylene propylene diene monomer (EPDM) rubber with varying content of carbon black under three different test configurations. Shen et al. [10,11] comparatively investigated the fretting wear behavior of acrylonitrile–butadiene rubber (NBR) under different working conditions. Guo et al. [12] described an experimental study on the fretting wear behavior and tribological mechanism between rubber and concrete in the process of pipeline laying. Zhou et al. [13] studied the fretting wear behavior of the rubber O-ring seal considering the effect of hydrogen swelling.

Although the micron-level fretting wear and contact of rubber materials were rarely reported, studies on ceramics and metals have been extensively developed. Mindlin [14] proposed that there were slip regions and non-slip regions in the fretting contact area under certain conditions and analyzed the fretting stress distribution on the contact surface. Waterhouse [15] divided the fretting process into three stages: initial, oxidation and steady states. Many studies have shown that frequency [16], normal force [17,18], and displacement amplitude [19,20] were important influence factors on the wear behavior of materials. Campbell et al. [21] investigated the fretting wear behavior of selected ceramics and cermets using a ball-on-disc fretting wear tester. Shu et al. [22,23] presented the experimental investigation on the fretting wear behavior of piezoceramics and piezoelectric thin films against Si_3_N_4_ ceramic ball under air condition. The influences of the displacement amplitude, frequency, normal force, and applied voltage were taken into account. Yuan et al. [24] discussed the fretting running behavior and damage mechanisms of Cu–Mg alloy in different fretting regions in detail. Xin et al. [25] investigated the evolution of fretting wear behavior and damage mechanism in Alloy 690TT. Duan et al. [26] identified the role of nitrides (converted carbides) in fretting wear characteristics through the fretting experiments of the X210CrW12 steel against GCr15 steel ball. Zhang et al. [27] evaluated the microstructure and fretting wear behavior of the 25CrNi2MoV steel. Wang [28] studied the influence of wear debris on the fretting wear characteristics of the nitrided medium carbon steel under line contact conditions at elevated temperatures. In addition, many researchers studied the protective capabilities of different coatings in fretting wear. Xu et al. [29] studied three bonded solid lubrication coatings on AISI E4142 steel to compare their fretting wear behaviors and mechanisms. Lisa et al. [30] studied the fretting wear behavior of two Zn-Ni coatings with different surface morphologies under various contact conditions including the stick, mixed slip, and gross slip. Wu et al. [31] found that AT40 coating and Al_2_O_3_/AT40 composite coating significantly improved the fretting wear resistance of TC6 alloy. Sharma et al. [32] presented a two-dimensional (2D) plane strain finite element model to simulate the fretting wear of the composite cermet coating. Niu et al. [33] investigated the fretting wear mechanism of a plasma-sprayed CuNiIn coating on a Ti-6Al-4V substrate using a bench level test. Lin et al. [34] investigated the behavior of duplex chameleon/PEO coatings on an Al alloy substrate in fretting wear tests against different conditions. Furthermore, some recent works on the friction and wear of rubbers can refer to Bayrak and Paulkowski [35], Szczypinski-Sala and Lubas [36], Pan et al. [37], and Vaikuntam et al. [38].

In this paper, the fretting wear of EPDM (ethylene propylene diene monomer), FPM (fluororubber), and NBR (nitrile–butadiene rubber) are studied in experiments. The experiment data are recorded by the instrument for plotting the friction logs. The contact surface morphologies of rubbers are observed by using the 3D white-light interfering profilometer and scanning electron microscopy (SEM). Then, the effects of normal force, frequency, displacement amplitude, and rubber hardness on fretting wear behaviors of three kinds of rubber are discussed. The fretting wear performances of three rubbers are compared by considering the effect of the displacement amplitude, normal force, frequency, and rubber hardness.

This paper makes the first attempt to analyze micron-level fretting wear behaviors of rubber materials. The new aspects are highlighted as follows: (i) We compare the fretting wear performances of three rubbers under the effect of the displacement amplitude, normal force, frequency, and rubber hardness. (ii) The wear mechanism of the three rubbers is discussed. The forms of wear include adhesive wear, fatigue wear, and abrasive wear. (iii) It is found that the wear debris agglomerated at the contact center may form a local sticky layer or viscous film, which acts as a protective layer or lubricating film to prevent further damage of the rubber surface.

## 2. Materials and Experimental Procedure

### 2.1. Materials

Three kinds of rubbers (EPDM, FPM, and NBR) are considered in experiments. They are widely used in various fields as an important sealing material. The commercial names of EPDM, FPM, and NBR are ethylene propylene diene monomer, fluororubber, and nitrile–butadiene rubber, respectively. The rubber materials are provided by Hebei Fengshuo Rubber and Plastic Pipe Industry Co., Ltd., Hengshui, China. Three kinds of rubbers are synthetic high-elastic polymers. NBR is made by copolymerizing diene and acrylonitrile. FPM is a synthetic rubber that contains fluorine atoms in its molecular structure. EPDM is a terpolymer of ethylene, propylene, and non-conjugated diene. The material properties of rubbers are listed in Table 1.

The basic compositions of rubbers are listed in Table 2. Zinc oxide (ZnO) and stearic acid (SA) are used as the activator. Tetramethyl thiuram disulfide (TTD) is used as the accelerator. Carbon black (CB) of the type N330 is used as the filler. Sulfur (S) and vulcanizing agent (VA) are used as the curing agent. The hardness of the rubber can be adjusted by changing the compositions. Indeed, the rubber matrix reinforced by some fillers such as the carbon black can improve their performance in practical applications.

The spherical punch made of the 304 stainless steel is chosen as the upper specimen with the diameter of 6.0 mm, surface roughness *Ra* = 0.02 μm, Young’s modulus 200 GPa, density 7916 Kg/m^3^, and Poisson’s ratio 0.29. The lower specimen is the rubber on the S45C steel flat. The rubber sample has a diameter of 18 mm and a thickness of 0.5 mm. The length and width of the S45C steel flat are 25 mm, the thickness is 5 mm. The density of S45C steel is 7850 Kg/m^3^ and Young’s modulus is 210 GPa. The 707 quick-drying glue is used to bond rubber and S45C steel flat. After curing for 24 h, the lap shear strength of the adhesive is greater than or equal to 10 MPa. In the tests, we choose the original rubber samples with a thickness of 6 mm to measure their hardness. In fretting wear tests, we use the 0.5 mm thick rubber sheet bonded on the S45C steel flat. The durometer is Sanliang LX-A. The implementation standard of hardness measurement is ISO 7619-1:2004.

### 2.2. Experimental Procedure

**Figure 1:**
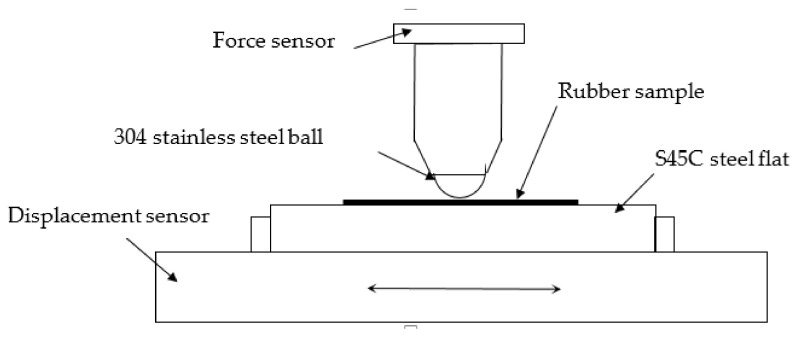
Schematic map of the fretting wear setup.

The fretting tests of rubber against steel ball are conducted in a reciprocating friction wear tester (Bruker UMT-TriboLab). The upper 304 stainless steel ball contacts on the rubber specimen under a prescribed normal force. After that, the horizontal displacement of the steel ball is fixed. The lower specimen (rubber on the S45C steel flat) is driven by a servo motor to make the reciprocating motion, as shown in Figure 1. It is noted that the normal force remains unchanged during the fretting test due to the automatic load control of a test machine.

**Figure 2:**
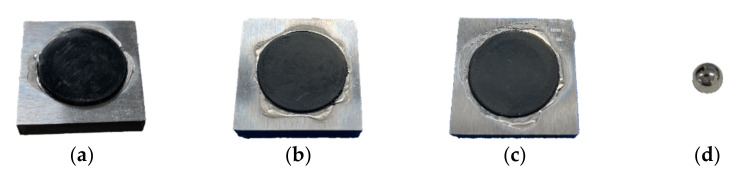
Fretting wear samples in the test: (**a**) EPDM; (**b**) FPM; (**c**) NBR; and (**d**) 304 stainless steel ball.

Before the experiment, the rubber samples and the steel sphere are washed in deionized water and acetone for 5 min with an ultrasonic cleaner. All fretting tests are displayed at room temperature 20 ± 2 °C and the humidity at 40%. The steel ball and rubber samples are shown in Figure 2. The experimental parameters are set as follows: imposed displacement amplitude *D* from ±0.1 mm to ±0.45 mm; normal force *F_n_* from 1 to 10 N; frequency *f* from 5 to 15 Hz, and number of cycles *N* from 1 to 10^4^ cycles. Moreover, for each case, three time tests are performed to average the final results.

**Figure 3:**
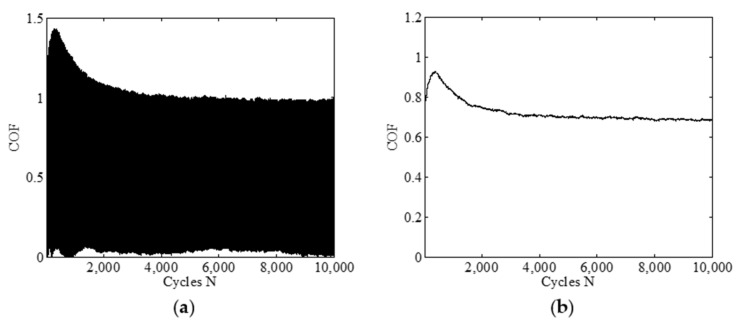
The friction coefficient of FPM under *F_n_* = 5 N, *d* = ±0.45 mm, *f* = 10 Hz, and *H* = 70 Ha: (**a**) original friction coefficient and (**b**) smoothed friction coefficient.

To explain how to obtain the friction coefficient, Figure 3 presents an example for calculating the friction coefficient of FPM under *F_n_* = 5 N, *d* = ±0.45 mm, *f* = 10 Hz and *H* = 70 Ha. The procedure is given as follows: (i) The data on the tangential force *F_t_* versus the instantaneous displacement are recorded as 50 points at each cycle. (ii) The friction coefficient *μ* is computed by real-time *F_t_*/*F_n_* at the stable sample points. Figure 3a shows the evolution of the original friction coefficient. (iii) The method of the Savitzky–Golay is adopted to smooth the friction coefficient, which is shown in Figure 3b. For details, please refer to Shu et al. [22] and Savitzky and Golay [39].

**Figure 4:**
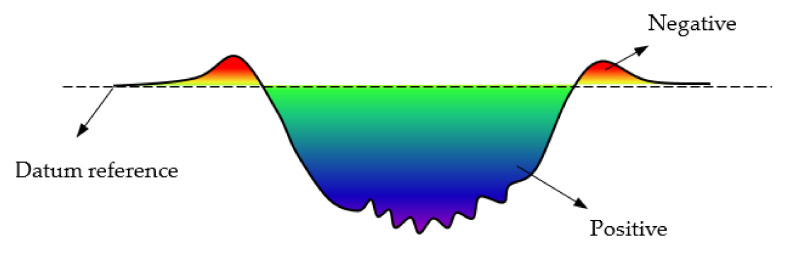
The sketch map for evaluating the wear volume.

Eventually, the wear scars of rubbers are detected by using SEM. The 3D white-light interfering profilometer (Zegage^TM^ Plus) is applied to characterize the surface profile. The wear volume is directly read from the software of Universal Profilometer UP-24 based on the 3D profile. Figure 4 gives the sketch map for evaluating the wear volume. The detailed procedure is given as follows: (i) Get the surface information by using the white light interference profilometer. (ii) Set the original surface as a datum reference and level the surface. (iii) Then, the software will run integral for all the points under datum reference and calculate the wear volume value. In addition, some scholars use other methods to characterize the wear volume. For example, Harea et al. [40] defined the wear volume by measuring the width of the track.

## 3. Test Results

### 3.1. Curves

The variation of the friction force (F_t_) versus relative displacement (D) as a function of the number of cycles (N) can be used to characterize the running state of the friction interface and the fretting behavior. During the fretting test, the following three basic shapes of *F_t_*—*D* curves can be obtained: straight, parallelogram, and ellipse. The straight and elliptic curves indicate that the two contact surfaces are in the state of slipping at the contact edge and sticking at the center, and the fretting process runs in the partial slip region. Among them, the elliptic curve is caused by the elastic hysteresis and plastic deformation of the rubber surface. The parallelogram shows the gross relative slip of the two contact surfaces, and the fretting process runs in the gross slip region. When there are two or more types of curves that convert each other, the fretting process runs in the mixed region. The area of the fretting loop represents the energy dissipation during the fretting process [41]. Note that the dissipated energy can be calculated, but it is tedious. Therefore, in the present paper, we only compare the dissipated energy of three rubbers from the fretting loop but do not calculate the practical value. The value of friction is a process that varies with the reciprocating displacement. Therefore, *F_t_*—*D* curves indicate the frictional sliding behaviors between a rubber plate and a steel ball. For details, please refer to Zhou et al. [42] for the explanation of *F_t_*—*D* curves.

**Figure 5:**
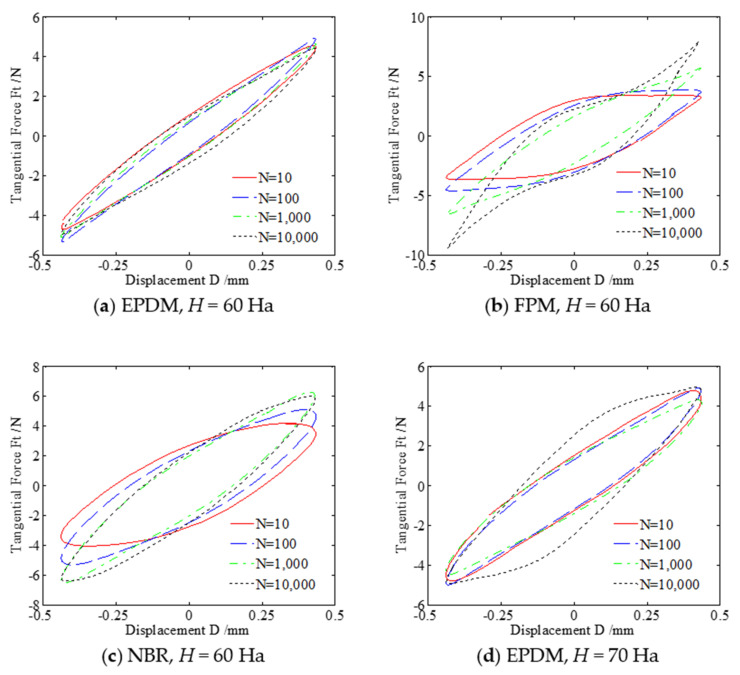

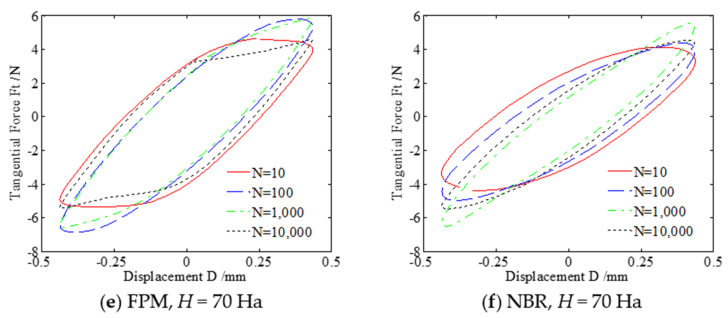
Curves of three rubbers under *F_n_* = 5 N, *d* = ±0.45 mm, and *f* = 10 Hz.

Figure 5a–c show *F_t_*—*D* curves of three rubbers under normal force *F_n_* = 5 N, displacement amplitude *d* = ±0.45 mm, frequency *f* = 10 Hz, and hardness *H* = 60 Ha. It can be seen from Figure 5a that curves of EPDM form the shape that is approximately linear in the entire fretting process. The displacement is mainly coordinated by the elastic deformation of the rubber itself. The maximum friction force is almost constant, and the fretting process runs in the partial slip region. As shown in Figure 5c, the tangential force of NBR gradually increases with the increase of the number of cycles. The elliptic *F_t_*—*D* curve indicates that there is energy dissipation during the fretting process, and the fretting process runs in the partial slip region. However, in Figure 5b, the tangential force has an abrupt change after about 1000 cycles, which indicates that the wear debris is suddenly peeled off, and the surface of FPM rubber is severely worn. The *F_t_*—*D* curve transitions from a parallelogram shape to an ellipse shape, and finally, it becomes a quasi-parallelogram shape with sharp corners. The fretting process runs in the mixed region.

Figure 5d–f show *F_t_*—*D* curves of three rubbers under normal force *F_n_* = 5 N, displacement amplitude *d* = ±0.45 mm, frequency *f* = 10 Hz, and hardness *H* = 70 Ha. Note that the hardness increases from 60 Ha in Figure 5a–c to 70 Ha in Figure 5d–f. Compared with Figure 5b, it is found in Figure 5e that the maximum tangential force of FPM gradually decreases from about 10 to 7 N and the sharp corners of the *F_t_*—*D* curve disappear. This is because the increase of hardness leads to the decrease of adhesion and hysteresis resistance between the FPM rubber and the metal, which makes the relative slip easier. However, compared with Figure 5a,c, the maximum tangential force and the curve shape of EPDM and NBR do not change significantly, as shown in Figure 5d,f.

### 3.2. Friction Coefficient Curves

**Figure 6:**
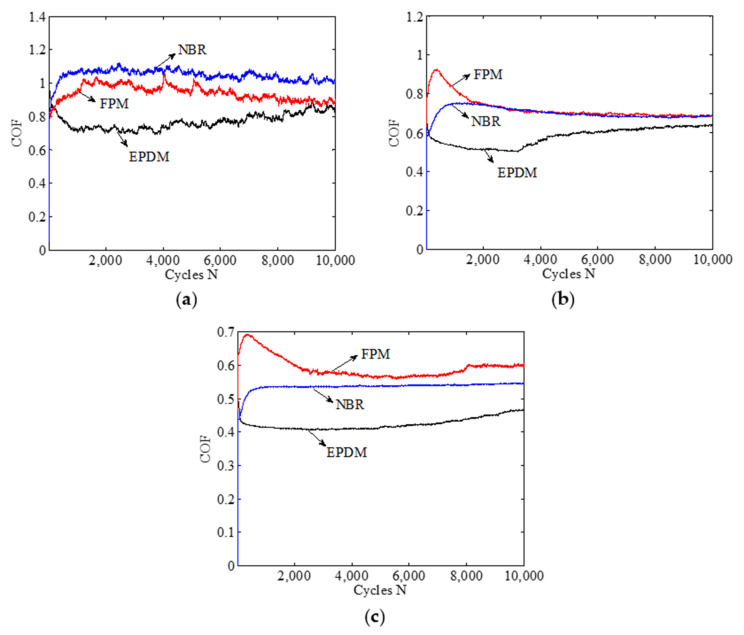
The friction coefficient curve of three rubbers under *d* = ±0.45 mm, *f* = 10 Hz, and *H* = 70 Ha: (**a**) *F_n_* = 1 N; (**b**) *F_n_* = 5 N; and (**c**) *F_n_* = 10 N.

Figure 6 shows the friction coefficient curves of three rubbers under different normal forces (1 N, 5 N, 10 N). The other loading conditions are set as *d* = ±0.45 mm, *f* = 10 Hz, and *H* = 70 Ha. In special, the friction coefficient of rubbers is great, and its value is about 0.4–1.05. The friction coefficient of rubbers is greater than that of metals and ceramics under the same experimental conditions. The reason is that the surface of rubbers may yield large deformation during the wear process, which in turn leads to a greater friction coefficient. With the increase of the normal force, the increase rate of the total contact area is less than that of the normal force, which leads to the decrease of the friction coefficient [5]. In Figure 6a, for a small normal force (*F_n_* = 1 N), the friction coefficient of three rubbers fluctuates markedly, which is caused by the discontinuous contact of the friction pair as the wear debris on the rubber surface acts as the third body. In Figure 6b,c, the friction coefficients of FPM and NBR increase rapidly at the initial stage and then gradually fall to a stable value. However, the friction coefficients of NBR increase rapidly at the first few cycles and then reach a stable value without the decreasing trend. For all three rubbers, increasing the normal force from 1 to 10 N will lead to a significant decrease of the friction coefficient.

**Figure 7:**
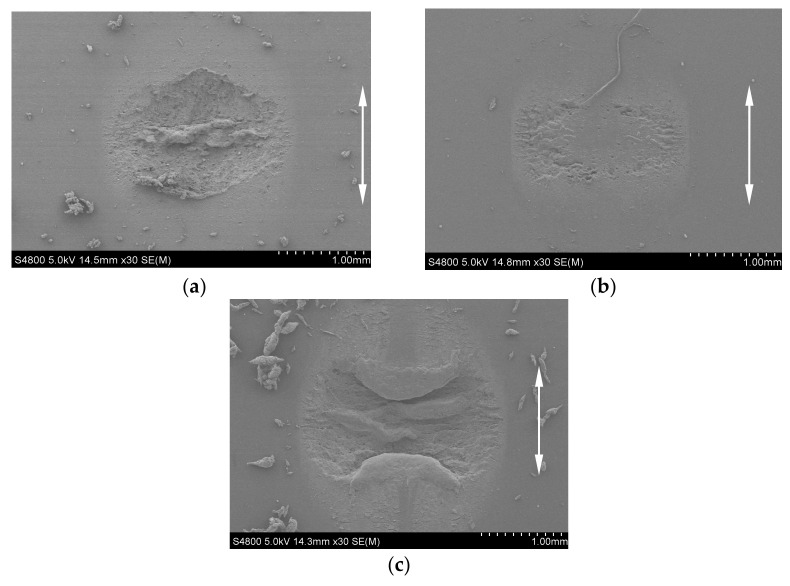
The SEM morphology of three rubbers under *F_n_* = 10 N, *d* = ±0.45 mm, *f* = 10 Hz, *H* = 70 Ha, and *N* = 10^5^ cycles: (**a**) FPM; (**b**) NBR; and (**c**) EPDM.

To explain why FPM has the maximum friction coefficient in Figure 6c, Figure 7 shows the SEM morphologies of a surface feature of three rubbers under *F_n_* = 10 N, *d* = ±0.45 mm, *f* = 10 Hz, *H* = 70 Ha, and *N* = 10^5^ cycles. The reasons of Figure 6a,b are similar to that of Figure 6c and thus will not be given for brevity. Obviously, among the three rubbers, the surface wear of EPDM is most serious, while that of NBR is slightest. However, as for EPDM, we can observe that a large amount of wear debris connected with the rubber remains at the contact region and acts as the solid lubricant [6]. Therefore, the rough worn surface leads to the highest friction coefficient of FPM, and the solid lubricant of wear debris leads to the smallest friction coefficient of EPDM.

**Figure 8:**
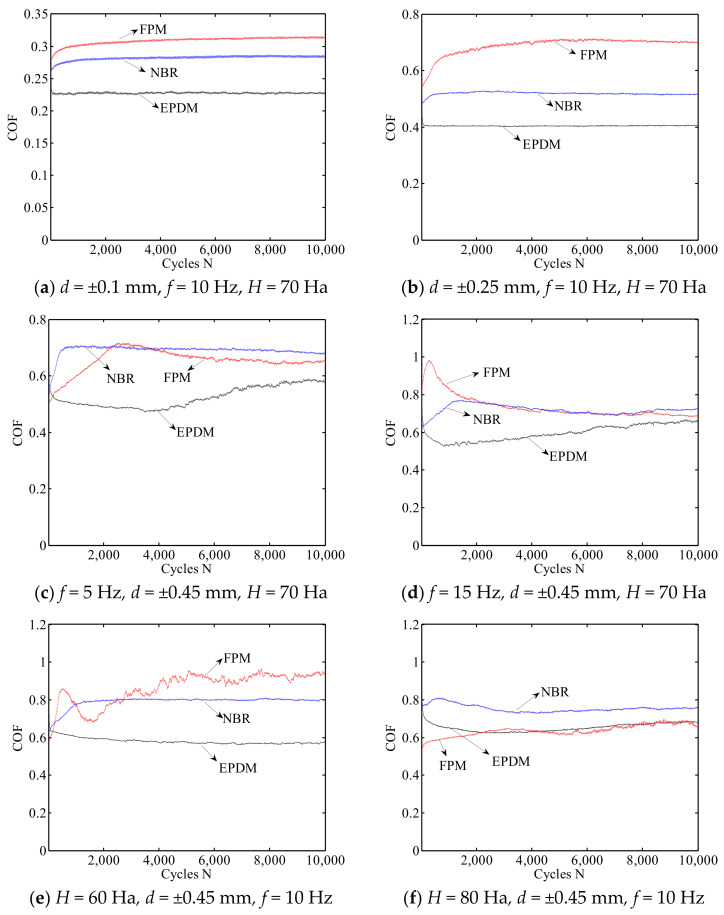
The friction coefficient curve of three rubbers under *F_n_* = 5 N.

Figure 8a,b show the friction coefficient curve of three rubbers for different displacement amplitudes (±0.1 mm, ±0.25 mm, ±0.45 mm) under *F_n_* = 5 N, *f* = 10 Hz, and *H* = 70 Ha. Note that the result *d* = ±0.45 mm is given in Figure 6b and is not shown here for brevity. The friction coefficient of all rubbers increases with the increase of the displacement amplitude. For a relatively small displacement amplitude (*d* = ±0.1 mm, ±0.25 mm), there is no relative slip between the two contact bodies, and the friction coefficient of three rubbers quickly reaches a stable value after several cycles. The friction coefficient of EPDM is the smallest, while that of FPM is the largest. However, for a relatively large displacement amplitude (*d* = ±0.45 mm), only the friction coefficient of NBR can quickly reach a stable value.

Figure 8c,d show the friction coefficient curve of three rubbers for different frequencies (5 Hz, 10 Hz, 15 Hz) under *F_n_* = 5 N, *d* = ±0.45 mm, and *H* = 70 Ha. Note that the result *f* = 10 Hz is given in Figure 6b. For all three frequencies, the friction coefficients of FPM and NBR are quite close at the stable stage, and their values are greater than those of EPDM. In Figure 8c, at the initial stage (about 1 to 2000 cycles), the friction coefficient of EPDM drops rapidly from the maximum value, while that of FPM first increases and then falls sharply from 100 to 2000 cycles. After about 4000–6000 cycles, the friction coefficients of three rubbers reach the stable stage, which means that the deformation and wear of the contact surface change slightly. With the increase of frequency, the friction coefficients of the three rubbers show a slightly increasing trend.

Figure 8e,f show the friction coefficient curve of three rubbers for different hardness (60 Ha, 70 Ha, 80 Ha) under *F_n_* = 5 N, *d* = ±0.45 mm and *f* = 10 Hz. The result of *H* = 70 Ha is given in Figure 6b. In Figure 8e with a small hardness (*H* = 60 Ha), we can see that the friction coefficient of FPM is maximum and shows an obvious oscillation. The friction coefficients of EPDM and NBR are relatively stable. The reason for the oscillation in FPM is because the wear debris is generated, and the peeling layer accumulates on the surface of FPM at the fretting direction. This makes it difficult to discharge the wear debris. This phenomenon can be observed in the corresponding SEM images. In Figure 8f with a large hardness (*H* = 80 Ha), the degree of cross-linking of rubber increases, and it is not easily damaged by mechanical stress. The friction coefficient of the three rubbers tends to be stable.

**Figure 9:**
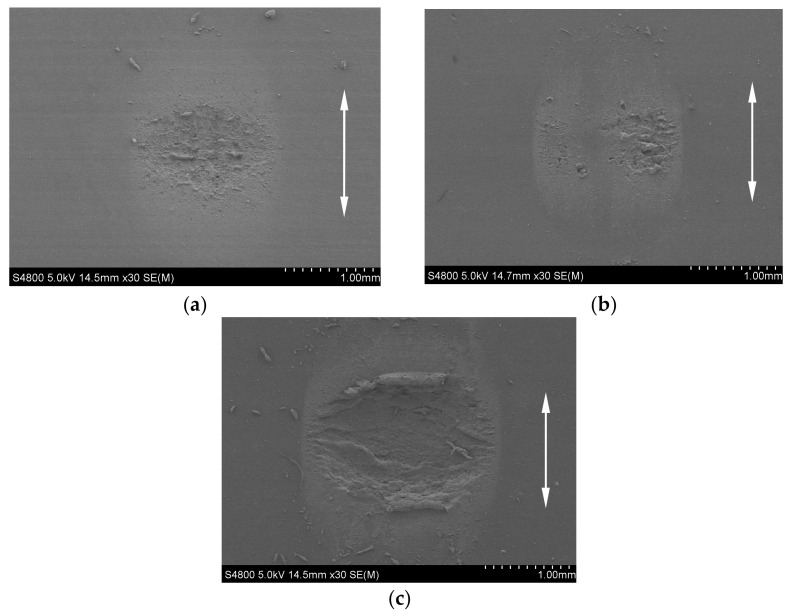
The SEM morphology of three rubbers under *F_n_* = 5 N, *d* = ±0.45 mm, *f* = 5 Hz, *H* = 70 Ha, and *N* = 10^5^ cycles: (**a**) FPM; (**b**) NBR; and (**c**) EPDM.

Figure 9 shows the SEM morphologies of a surface feature of three rubbers corresponding to Figure 8c. We can observe that the surface wear damages of FPM and NBR are comparable, and they are milder than that of EPDM. However, as for EPDM, a large amount of wear debris remains at the contact region, and it acts as the solid lubricant. That is why the friction coefficients of FPM and NBR are quite close at the stable stage and their values are greater than those of EPDM.

### 3.3. Surface Morphology and Wear Mechanism

**Figure 10:**
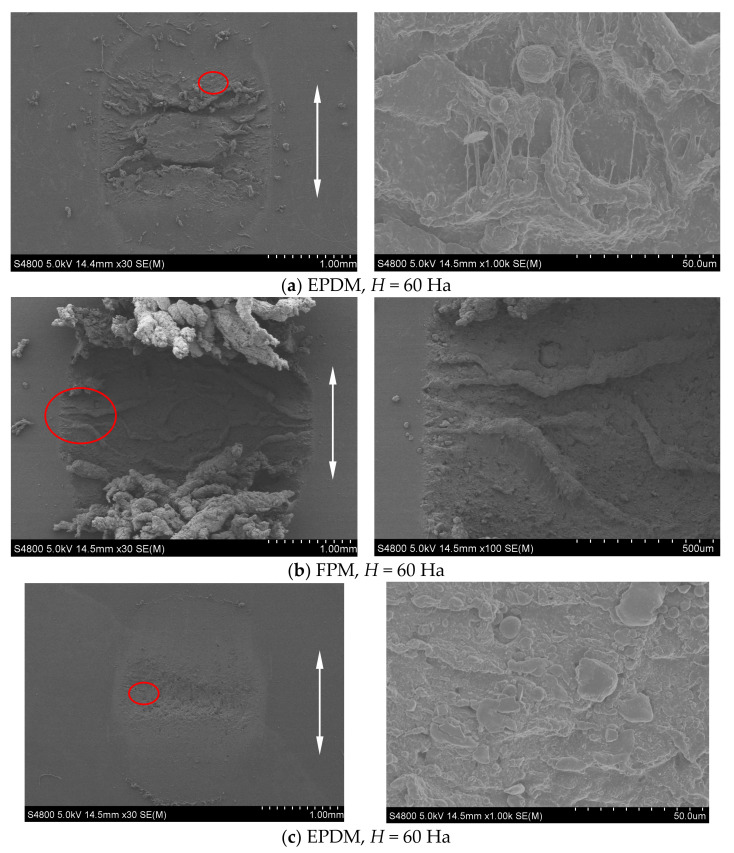

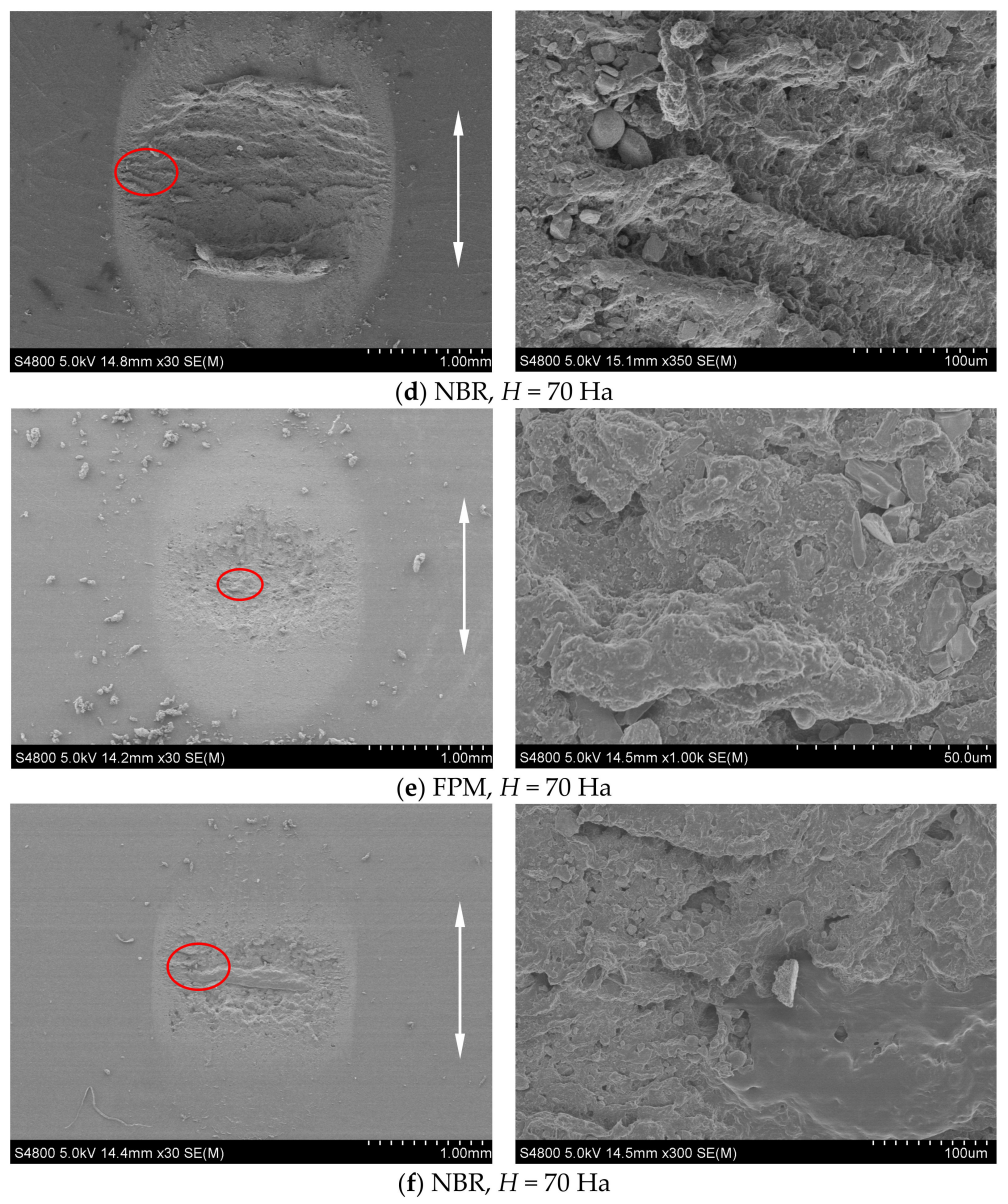
The SEM morphology of three rubbers under *F_n_* = 5N, *d* = ±0.45mm, and *f* = 10Hz (left: the whole contact region; right: the local magnification).

Figure 10a–c show the SEM morphologies of three rubbers under *F_n_* = 5 N, *d* = ±0.45 mm, *f* = 10 Hz, and *H* = 60 Ha. The left figure shows the whole contact region and the right one shows the local magnification of the wear scar at the location of the red mark. In Figure 10a, it can be observed that the fatigue cracks are generated between the central adhesion zone and the slip zone of EPDM, which then expand to form grooves. We can see the obvious wear in EPDM, including the periodic tearing to form the tongue, the fracture of the tongue root, tangential stress to form the curled wear debris, and gelatinous wire drawing. The forms of wear include adhesive wear, fatigue wear, and abrasive wear. The most severe wear is the FPM, as shown in the left of Figure 10b. We can find a large number of crimp chips on both sides parallel to the fretting direction, and the wear debris is not separated from the rubber. This is the result of the rubber surface rolling from the center to the sides under the action of the tangential force. The rolled-up state of the rubber surface can be seen in the right of Figure 10b, too. It is mainly adhesive wear and abrasive wear. Compared with EPDM and FPM, NBR has better abrasion resistance, as shown in Figure 10c. A large number of abrasive particles and fine scratches can be observed on the rubber surface, which is mainly the abrasive wear.

Figure 10d–f show the SEM morphology of three rubbers under *F_n_* = 5 N, *d* = ±0.45 mm, *f* = 10 Hz, and *H* = 70 Ha. Note that the hardness of the rubbers increases from 60 Ha in Figure 10a–c to 70 Ha in Figure 10d−f. In Figure 10d, the wear scar on the EPDM rubber surface is perpendicular to the fretting direction, and the wear scars form ridge-like protrusions on the surface, that is, Schallamach pattern wear [43]. At the same time, the curled wear debris is produced on the worn surface. In Figure 10e, there is no large curled wear debris. Compared with Figure 10b, the wear of FPM rubber is much less for a relatively large hardness (*H* = 70 Ha). In Figure 10f, the wear debris is agglomerated at the contact center of the NBR rubber to form a local sticky layer or viscous film, which acts as a protective layer or lubricating film to prevent further damage of the rubber surface [10].

### 3.4. Wear Volume

The 3D white-light interfering profilometer is an ideal non-contact tool for the quantitative measurement of wear profile and surface roughness. Surface wear volume can be obtained by using the built-in software for surface data image processing.

**Figure 11:**
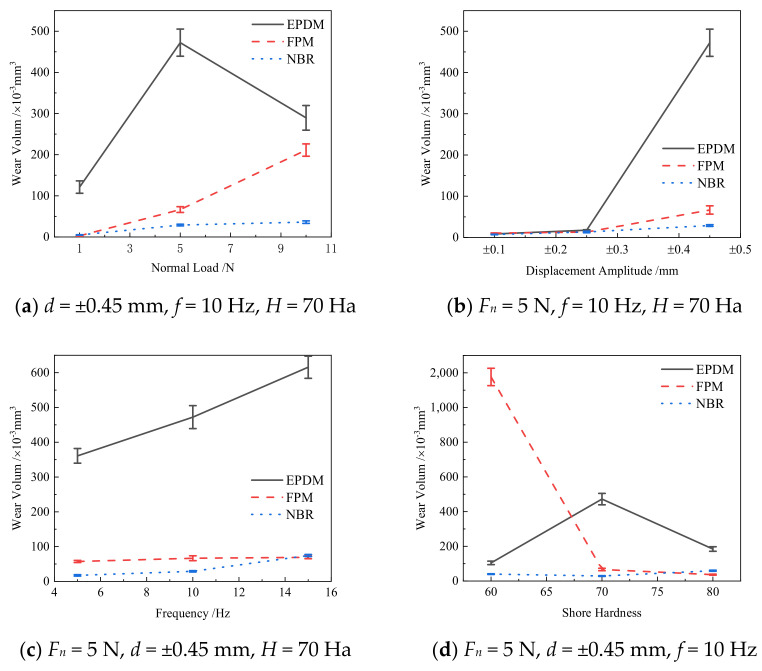
The wear volume of three rubbers.

Figure 11a shows the relationship between the wear volume and normal force of the three rubbers under *d* = ±0.45 mm, *f* = 10 Hz, and *H* = 70 Ha. The wear volume of EPDM is the largest of the three rubbers, and it first increases and then decreases with the increasing normal force. However, the wear volume of FPM and NBR increases with the increase of the normal force. As shown in Figure 7c, for a large normal force (*F_n_* = 10 N), severe plastic deformation occurs on the surface of EPDM. The contact region of EPDM is squeezed from both sides to the center, which makes the wear volume under *F_n_* = 10 N smaller than that under *F_n_* = 5 N.

Figure 11b shows the relationship between the wear volume and displacement amplitude of three rubbers under *F_n_* = 5 N, *f* = 10 Hz, and *H* = 70 Ha. The wear volume increases with the increase of displacement amplitude. When the displacement amplitude is small, the displacement is completely adjusted by the elastic deformation of the contact surface, and the area of the adhesive region is much larger than that of the micro-slip region. The wear volume of the three rubbers is almost the same.

Figure 11c shows the relationship between the wear volume and frequency of three rubbers under *F_n_* = 5 N, *d* = ±0.45 mm and *H* = 70 Ha. With the increase of frequency, the movement speed of the grinding chips is accelerated, and it is very easy to overflow the contact surface, which leads to the reduction of the grinding chips in the contact region, and the protective effect on the substrate is also reduced. This eventually leads to more severe wear and tear. In addition, the wear volume of EPDM is still the largest.

Figure 11d shows the relationship between the wear volume and rubber hardness of three rubbers under *F_n_* = 5 N, *d* = ±0.45 mm and *f* = 10 Hz. The wear volume of NBR rubber is always kept at a relatively small value. With the increasing hardness, the wear volume of FPM rubber decreases, while the wear volume of EPDM rubber decreases first and then increases.

It should be pointed out that the fretting wear behavior of rubbers may be affected by many factors, such as displacement amplitude, normal force, frequency, and rubber hardness. We can only judge which one is best in the given condition.

## 4. Conclusions

Investigations on the fretting wear behavior of three kinds of rubbers (EPDM, FPM, NBR) against a steel ball are conducted through a sphere-on-flat contact configuration. The fretting wear behavior, damage characteristic, and wear mechanism are discussed in details. According to the results of the fretting wear tests, we can find that for all three rubbers, the fretting regions (partial slip, mixed, and gross slip) depend on the displacement amplitude, frequency, normal force, rubber hardness, and number of cycles. The friction coefficient of three rubbers decreases with the increase of the normal force, but it increases with the increase of the displacement amplitude. In particular, EPDM rubber has the lowest friction coefficient among the three rubbers. The maximum tangential force and friction coefficient of FPM rubber decrease with the increase of hardness. However, the maximum tangential forces and friction coefficients of EPDM and NBR are not sensitive to the rubber hardness. NBR rubber has the best wear resistance among the three rubbers, while EPDM rubber has the worst wear resistance under large displacement and high frequency, and FPM has the worst wear resistance under small hardness and large normal force.

## Figures and Tables

**Table 1:** Material properties of rubber samples.

Properties	EPDM	FPM	NBR
Hardness, *H* (Ha)	60–80	60–80	60–80
Young’s modulus, *E* (MPa)	6.65–16.53	3.44–16.06	3.41–16.75
Surface roughness, *Ra* (μm)	0.23–0.27	0.21–0.25	0.17–0.30

**Table 2:** Basic compositions of EPDM, FPM, and NBR rubbers (phr—parts per hundred rubber).

Rubber.	ZnO (phr)	SA (phr)	TTD (phr)	S (phr)	VA (phr)	CB (phr)
EPDM (*H* = 60 Ha)	5	1	1.5	1.5	-	24
FPM (*H* = 60 Ha)	5	1	-	-	3.0	26
NBR (*H* = 60 Ha)	5	1	1.5	1.5	-	24
EPDM (*H* = 70 Ha)	5	1	1.5	1.5	-	44
FPM (*H* = 70 Ha)	5	1	-	-	3.0	46
NBR (*H* = 70 Ha)	5	1	1.5	1.5	-	44
EPDM (*H* = 80 Ha)	5	1	1.5	1.5	-	64
FPM (*H* = 80 Ha)	5	1	-	-	3.0	66
NBR (*H* = 80 Ha)	5	1	1.5	1.5	-	64

## Data Availability

All data, models, or code generated or used during the study are available from the corresponding author by request.
